# Emodin combined with 5‐aminolevulinic acid photodynamic therapy inhibits condyloma acuminate angiogenesis by targeting SerRS

**DOI:** 10.1111/jcmm.70122

**Published:** 2024-10-01

**Authors:** Hongyan Lu, Zhangsong Peng, Yingrui Luo, Zhaohui Zheng, Changxing Li, Qi Wang, Chao Han, Youyi Wang, Liuping Liang, Kang Zeng, Yuxiang Chen

**Affiliations:** ^1^ Department of Dermatology and Venereology, Nanfang Hospital Southern Medical University Guangzhou China; ^2^ Department of Plastic Surgery, Nanfang Hospital Southern Medical University Guangzhou China; ^3^ School of Basic Medical Sciences Southern Medical University Guangzhou China; ^4^ The Second Affiliated Hospital of Zhejiang Chinese Medical University Xinhua Hospital of Zhejiang Province Hangzhou China

**Keywords:** condyloma acuminatum, emodin, photodynamic therapy, SerRS, VEGFA

## Abstract

Human papillomavirus (HPV) infection can cause condyloma acuminatum (CA), which is characterized by a high incidence and a propensity for recurrence after treatment. Angiogenesis plays an important role in the occurrence and development of CA. Seryl‐tRNA synthetase (SerRS) is a newly identified, potent anti‐angiogenic factor that directly binds to the vascular endothelial growth factor (VEGFA) promoter, thereby suppressing its transcription. Emodin is a natural anthraquinone derivative that can promote SerRS expression. This study aimed to investigate the effects of emodin on CA and explore combined treatment strategies. The HPV‐infected cell line SiHa was treated with either DMSO, emodin, ALA‐PDT or a combination of emodin and ALA‐PDT. We observed the effects on cell proliferation, apoptosis and the SerRS‐VEGFA pathway. Our findings demonstrated that emodin targets angiogenesis through the SerRS‐VEGFA pathway, resulting in the inhibition of SiHa cell proliferation and promotion of apoptosis (*p* < 0.001). To verify the therapeutic effect of emodin combined with ALA‐PDT on HPV‐associated tumours in vivo, we established an animal xenograft model by subcutaneously inoculating mice with SiHa cells (*n* = 4). The results showed that the combination of emodin and ALA‐PDT significantly inhibited the expression of VEGFA to inhibit angiogenesis (*p* < 0.001), thus showing an inhibitory effect on tumour (*p* < 0.001). Furthermore, we determined that the mechanism underlying the decrease in VEGFA expression after emodin combined with ALA‐PDT in CA may be attributed to the promotion of SerRS expression (*p* < 0.001). The combination of emodin and ALA‐PDT holds promise as a novel therapeutic target for CA by targeting neovascularization in condyloma tissues.

## 1 INTRODUCTION

Human papillomavirus (HPV) is a circular double‐stranded DNA virus, approximately 8.0 kbp in size, that infects epithelial cells (keratinocytes). Over 200 HPV genotypes exist. Mostly, α‐genotypes infect the mucosal epithelium, whereas the β‐genotypes mainly infect the skin epithelium. Most HPV presents as a recessive infection or benign lesion, cleared by the immune system. However, HPV is a clinically significant disease agent worldwide, with high burden, causing persistent infection, easily relapsing, leading to epithelial dysplasia and neoplasia in high‐risk groups for anogenital mucosal HPV infection.^1^ Therefore, early intervention and active treatment of HPV‐associated skin neoplastic diseases are significant. Traditional HPV treatment includes physical therapy, chemotherapy and surgery. However, these methods have limitations such as high recurrence rates and local trauma.^2^, ^3^

Condyloma acuminatum (CA) is a sexually transmitted disease caused by low‐risk HPV6/11 infection, presenting clinically as papillary or warty anogenital lesions. The disease is characterized by rapid growth and easy recurrence in practice, likely related to local neovascularization. Most topical CA treatments aim to eliminate warts, but recurrence rates are as high as 67%.^4^ CA shares cancer‐like characteristics, rarely resolves spontaneously, often recurs after surgical excision or laser cauterization, and requires long‐term repeated treatment, adversely affecting patient physical and mental health.^5^, ^6^ Devising combination therapy for CA and understanding its development mechanism could improve efficacy and reduce recurrence.

Photodynamic therapy (PDT) is a promising therapeutic method with advantages of minimal trauma and low systemic toxicity.^7^ Due to its potent cytotoxic effect on anorectal warts, PDT has become one of the most promising strategies for CA.^8^, ^9^, ^10^ 5‐aminolevulinic acid (5‐ALA) is a new type of second‐generation photosensitizer.^11^, ^12^ When PDT is used to treat CA, 5‐ALA can be taken up by cells with vigorous proliferation to generate a large amount of Protoporphyrin IX (PpIX), which mediates energy transfer from light to molecular oxygen and then produces reactive oxygen species.^13^ These reactive oxygen species have a very short half‐life and diffusion radius, rapidly triggering cytotoxic effects and causing cell death.^14^, ^15^ However, the mechanism of ALA‐PDT in treating CA has not been fully clarified and may include promoting keratinocyte apoptosis, regulating the immune response and damaging CA‐associated blood vessels.^16^, ^17^ Currently, there is a lack of studies on the effect of ALA‐PDT on CA tissue angiogenesis.

Dermal capillary hyperplasia and dilatation are key pathological changes in CA. PDT has a clear effect on blood vessels, damaging neovascularization in tissues and inhibiting proliferation of vascular endothelial cells. Vascular endothelial growth factor (VEGF) refers to both the originally identified dimeric glycoprotein now termed VEGFA, and the family of VEGF‐related polypeptides, that is, VEGFA, VEGFB, VEGFC, VEGFD and placental growth factor (PLGF).^18^, ^19^ The VEGF‐related pathway is closely associated with formation and maturation of new vessels, where VEGFA has a particularly significant effect on promoting angiogenesis and increasing vascular permeability. Studies show VEGFA expression is significantly enhanced in HPV‐infected cell lines and CA tissues, suggesting high VEGFA expression promotes CA occurrence and development.^20^ Kawczyk‐Krupka showed VEGF expression was significantly decreased in the ALA‐PDT treatment group versus control group with obvious anti‐angiogenesis effects.^21^ These results suggest PDT treatment can damage blood vessels at lesions in multiple ways and inhibit new blood vessel formation to some extent, inhibiting wart proliferation and disease progression. However, the exact mechanism by which ALA‐PDT inhibits VEGFA remains elusive.

Our previous study revealed a novel signalling cascade controlling VEGFA expression upon UV exposure, mediated by the ATM‐SerRS pathway.^22^ Seryl‐tRNA synthetase (SerRS) is a newly identified, potent anti‐angiogenic factor directly binding the VEGFA promoter to suppress transcription.^23^, ^24^, ^25^ Based on this, previous literature has screened small molecules from a 330‐compound traditional Chinese medicine library for ability to activate SerRS expression. They found emodin significantly increases SerRS expression, thereby decreasing VEGFA transcription.^26^

Emodin (1,3,8‐trihydroxy‐6‐methyl‐anthraquinone) is a natural anthraquinone derivative extracted from *rhubarb*, *polygonum cuspidatum*, *polygonum multiflorum* and other Chinese herbs.^27^ Emodin has a wide range of pharmacological properties, including anticancer, anti‐inflammatory, antioxidant and antibacterial effects.^28^, ^29^ In recent years, emodin has become increasingly popular due to its fewer side effects compared to standard cancer drugs.^30^ A review of the antiviral effects of emodin in recent decades indicates that it inhibits infection and replication of more than 10 viruses in vivo and in vitro.^31^, ^32^ Additionally, the latest research provides evidence suggesting that emodin and aloe‐emodin mediated PDT has potential for clinical development as a new effective and safe photosensitizer to treat skin cancer.^33^

The efficacy of PDT is excellent, but it does not fundamentally solve the problem of relapse, and relapse is related to HPV and neovascularization. In light of the limitations of current treatments and the need for novel therapeutic strategies, our study delves into the potential of emodin as an adjunct to ALA‐PDT. By exploring the role of SerRS in modulating VEGFA expression and its implications on angiogenesis, we aim to uncover a new avenue for the treatment of CA. The integration of emodin with ALA‐PDT may offer a synergistic approach that not only addresses the immediate cellular proliferation but also targets the underlying angiogenic processes that contribute to the recurrence of CA. Understanding these mechanisms is crucial for the development of more effective therapies with lower recurrence rates, ultimately improving patient outcomes and quality of life. This research, therefore, not only contributes to the existing body of knowledge but also paves the way for future clinical investigations into the combined use of traditional medicine and modern photodynamic techniques in the management of HPV‐associated diseases.

## 2 RESULTS

### 2.1 Expression of VEGFA in CA tissue was significantly higher than in healthy control tissue

**FIGURE 1:**
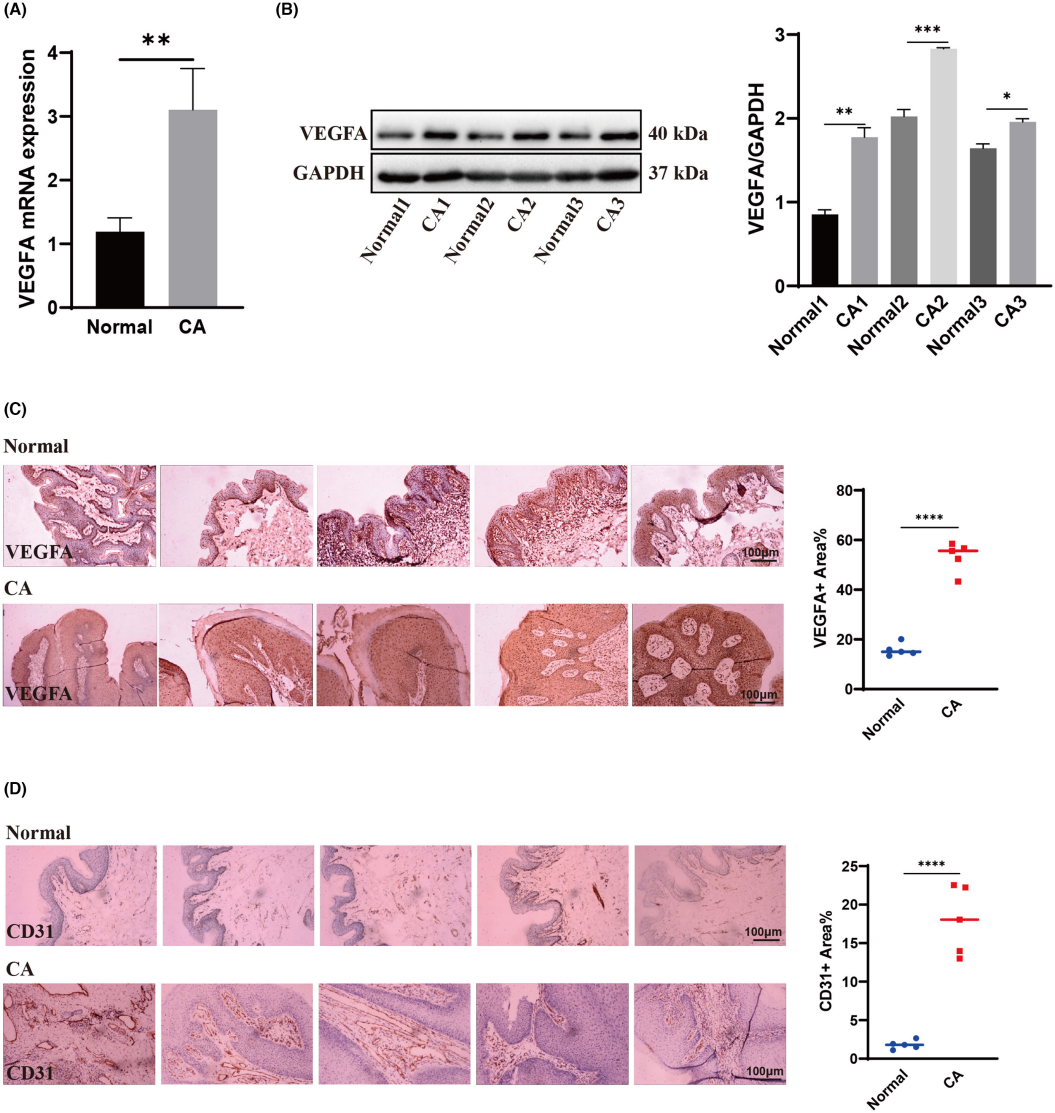
The expression of VEGFA in CA tissue was significantly higher than that in healthy control group. (A) Real‐time PCR showed the expression of VEGFA in CA tissue and normal preputial tissue (*n* = 5, Student's *t* test). (B) Western blot displayed the expression of VEGFA in CA tissue and normal preputial tissue (*n* = 3). (C, D) Immunohistochemistry assay displayed the level of VEGFA (brown) and CD31 (brown) in CA tissue and the healthy control group (*n* = 5, Student's *t* test). All images were taken at 200× original magnification, Scale bar, 100 μm. All experimental data were independently repeated for 3 times to obtain the mean ± SD. *p* value, * < 0.05, ** < 0.01, *** < 0.001, **** < 0.0001 by Student's t test.

To determine whether VEGFA plays a role in CA and investigate its function in CA proliferation and recurrence, VEGFA expression in human CA tissue and normal preputial tissue was examined. Compared with normal preputial tissue, the expression level of VEGFA mRNA and protein in CA was significantly increased (Figure 1A,B). Immunohistochemical results also confirmed that expression of VEGFA in CA tissues was significantly higher than in healthy control tissues (Figure 1C). We also studied skin angiogenesis in CA and normal preputial tissue using immunohistochemical analysis of platelet/endothelial cell adhesion molecule‐1 (CD31, also known as PECAM1). The results showed that the vascular dermis in CA tissue was significantly increased (Figure 1D). These results suggest that such high expression of VEGFA promotes the occurrence and development of CA and is closely related to excessive proliferation of CA and formation of new blood vessels.

### 2.2 Emodin inhibited SiHa cell proliferation and promoted apoptosis via the SerRS‐VEGFA pathway

**FIGURE 2:**
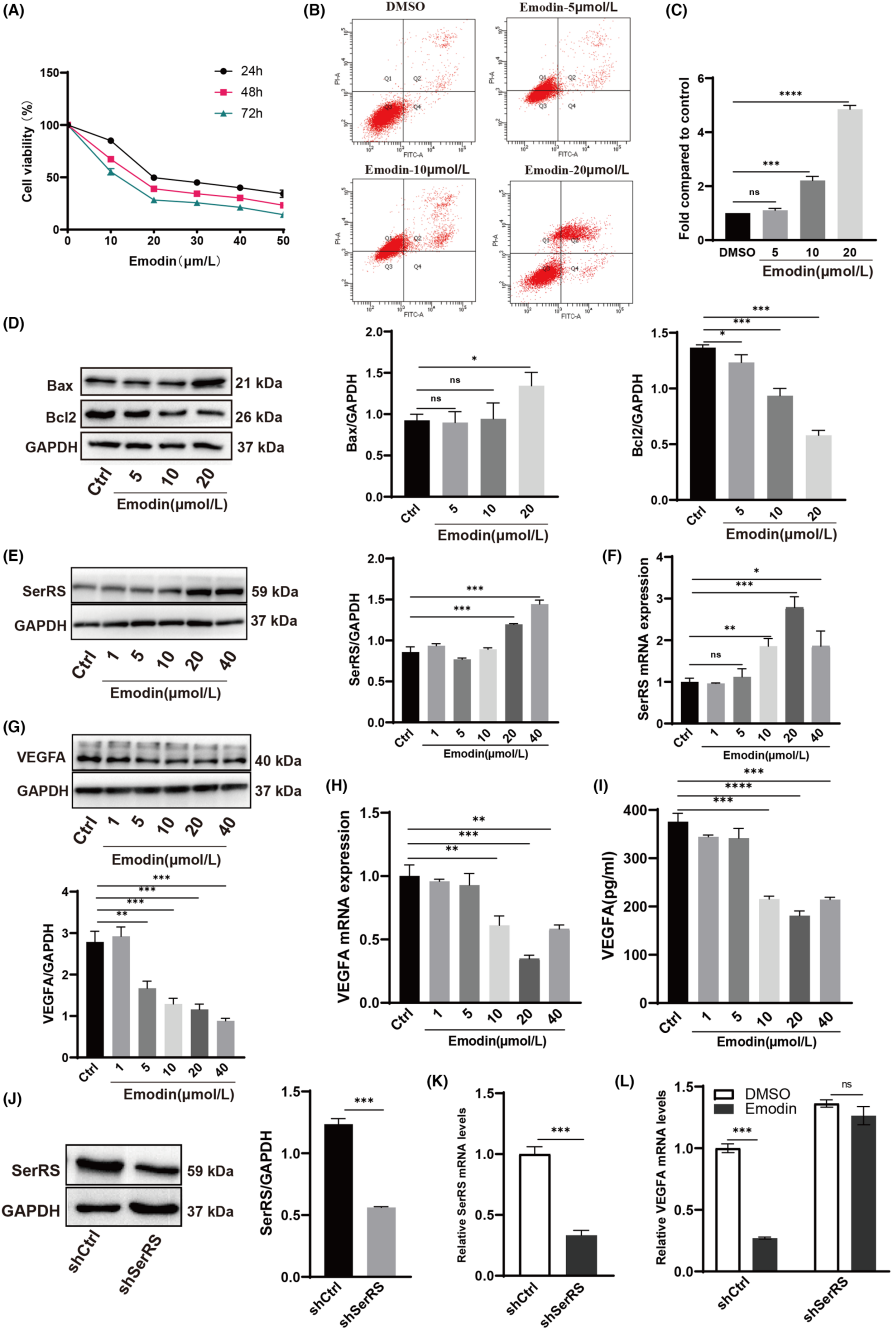
Emodin can inhibit proliferation and promote apoptosis of SiHa cells via the SerRS‐VEGFA pathway. (A) SiHa cells were treated for 24 h, 48 h and 72 h with emodin at 0, 10, 20, 30, 40 and 50 μmol/L concentrations, and SiHa cells viability was measured with CCK‐8 assay. (B, C) SiHa cells were treated for 24 h with emodin at 0, 5, 10 and 20 μmol/L concentrations and SiHa cells apoptosis rate was measured with Flow cytometry assay. (D) After different concentrations emodin treated SiHa cells 24 h, the apoptosis marker was detected with Western blot. (E, G) Western blot analysis displayed the expression of SerRS and VEGFA in SiHa cells treated with 1, 5, 10, 20 and 40 μmol/L emodin. (F, H) Real‐time PCR showed the level of SerRS and VEGFA in SiHa cells treated with 1, 5, 10, 20 and 40 μmol/L emodin. GAPDH was used as a loading control. (I) ELISA experiment revealed that the level of VEGFA in SiHa cells treated with different concentrations of emodin. (J, K) Western blot and Real‐time PCR experiments were conducted to assess the knockdown efficiency of SerRS. (L) Real‐time PCR was performed to evaluate the mRNA expression levels of VEGFA in the control group and the SerRS knockdown group after treatment with Emodin. All experimental data were independently repeated for 3 times to obtain the mean ± SD. *p* value, * < 0.05, ** < 0.01, *** < 0.001, **** < 0.0001 by Student's *t* test.

SiHa cells are high‐risk HPV16 whole‐genome human derived cells, and SiHa cells were used as a model of condyloma acuminatum to observe the effect of emodin. The cells were treated for 24 h, 48 h and 72 h with emodin at concentrations of 0, 10, 20, 30, 40 and 50 μmol/L, and cell viability was measured using the CCK‐8 assay. We found that emodin had a high cytotoxic effect against SiHa cells; the proliferation rate of SiHa cells decreased with increasing emodin concentrations and incubation times (Figure 2A). Thus, to a certain extent, emodin inhibited SiHa cell proliferation. Therefore, we used a concentration of emodin of 20 μmol/L and a culture time of 24 h in ensuing experiments. Flow cytometry results demonstrated that emodin promoted apoptosis of SiHa cells in a dose‐dependent manner (Figure 2B,C). Western blot results also confirmed that emodin could promote the increase of apoptosis marker protein Bax and the decrease of Bcl2 (Figure 2D), indicating that emodin inhibited SiHa cell proliferation and promoted SiHa cell apoptosis.

To explore the molecular pathway activated by emodin, we performed qRT‐PCR of VEGFA and SerRS and WB analysis using SerRS and VEGFA antibodies in cells treated with emodin. Cells were treated for 24 h with emodin at concentrations of 1, 5, 10, 20 and 40 μmol/L. We found an increase in SerRS level in SiHa cells treated with different concentrations of emodin (Figure 2E,F). In addition, a significant decrease in VEGFA level was observed in these cells (Figure 2G,H). ELISA experiment of VEGFA was also performed. Consistent with our expectation, VEGFA secretion was significantly reduced in emodin treated groups (Figure 2I).

To further confirm whether emodin‐induced VEGFA expression inhibition is caused by SerRS activation, we knocked down SerRS using shRNA (Figure 2J,K). qRT‐PCR results showed that emodin's inhibitory effect on VEGFA was abolished in SiHa cells with silenced SerRS (Figure 2L), indicating that emodin‐induced SerRS expression activation is necessary to block VEGFA.

### 2.3 Emodin enhanced the effect of ALA‐PDT, inhibited proliferation and promoted apoptosis in SiHa cells

**FIGURE 3:**
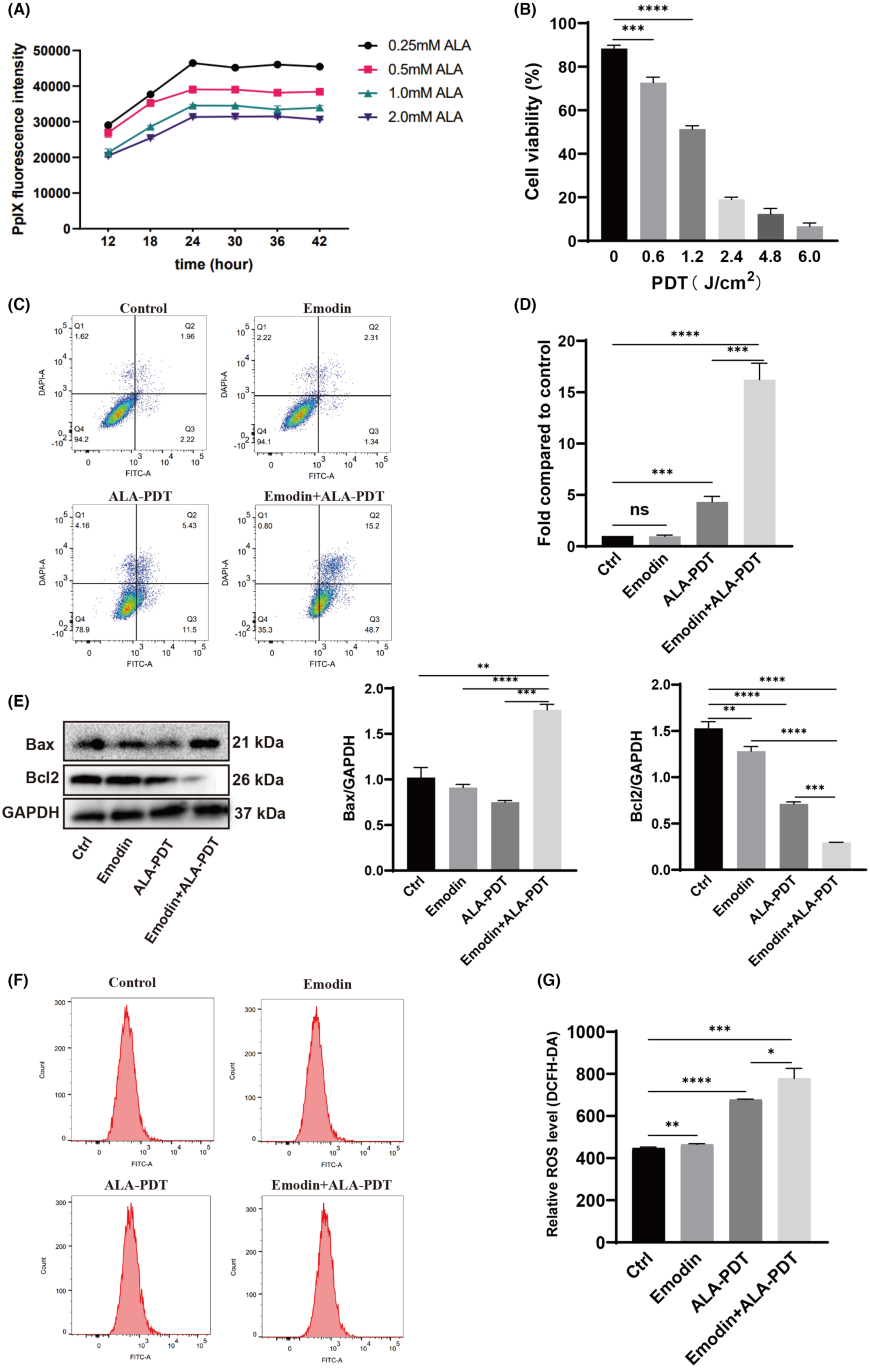
Emodin enhanced the effect of ALA‐PDT inhibited the proliferation and promoted the apoptosis of SiHa cells. (A) SiHa cells were treated for 12, 18, 24, 30, 36 and 42 h with ALA at 0.25, 0.5, 1.0 and 2.0 mmol/L concentrations and fluorescence intensity of PpIX was measured by BMG multifunctional enzyme marker. (B) SiHa cells were treated for 24 h with 0.25 mmol/L ALA, then disposed to PDT at the energy of 0, 0.6, 1.2, 2.4, 4.8 and 6.0 J/cm^2^. SiHa cell viability was measured using the CCK‐8 assay. (C, D) SiHa cells were treated for 24 h with emodin combined with ALA‐PDT and the apoptosis was assessed by flow cytometry using Annexin V/propidium iodide staining. (E) The apoptosis marker of Bax and Bcl2 were detected with Western blot. (F, G) The reactive oxygen species (ROS) production was measured in SiHa cells which were treated with emodin and ALA‐PDT or not by flow cytometry. All experimental data were independently repeated for 3 times to obtain the mean ± SD. *p* value, * < 0.05, ** < 0.01, *** < 0.001, **** < 0.0001 by Student's *t* test.

ALA is applied clinically in PDT for tumour treatment or imaging‐guided surgery. Its side effects are reported to be minimal, and it is clinically safe for cancer treatment. We cultured SiHa cells with ALA at concentrations of 0.25, 0.5, 1.0 and 2.0 mmol/L for 0, 12, 18, 24, 30, 36 and 42 h, and the fluorescence intensity of PpIX was measured by the BMG multifunctional enzyme marker. We found that the optimal concentration and treatment time of ALA were 0.25 mmol/L and 24 h, respectively (Figure 3A). Then, we cultured SiHa cells with ALA at 0.25 mmol/L. After 24 h, we treated the cells with PDT at energies of 0, 0.6, 1.2, 2.4, 4.8 and 6.0 J/cm^2^, and cell viability was measured using the CCK‐8 assay. As shown in Figure 3B, SiHa cell viability was significantly reduced at 1.2 J/cm^2^.

To determine whether emodin can affect the proliferation of SiHa cells in combination with ALA‐PDT, we treated SiHa cells with 0.25 mmol/L ALA for 24 h followed by 1.2 J/cm^2^ PDT (as was used in experiments described below) and then cultured them with medium containing 20 μmol/L emodin (as was used in experiments described below). Apoptosis was assessed by flow cytometry using annexin V/propidium iodide staining. As shown in Figure 3C,D, the number of apoptotic SiHa cells increased significantly when treated with emodin combined with ALA‐PDT compared with ALA‐PDT monotherapy. The results of Western blot further confirmed that emodin could enhance the apoptosis promoting effect of ALA‐PDT on SiHa cells (Figure 3E).

Flow cytometry measurements showed that reactive oxygen species production was significantly higher in emodin and ALA‐PDT‐treated SiHa cells compared to ALA‐PDT‐treated cells (Figure 3F,G). These findings indicate that emodin combined with ALA‐PDT had a stronger effect on SiHa cells.

### 2.4 Emodin enhanced the effect of ALA‐PDT on SiHa cells through the SerRS‐VEGFA pathway

**FIGURE 4:**
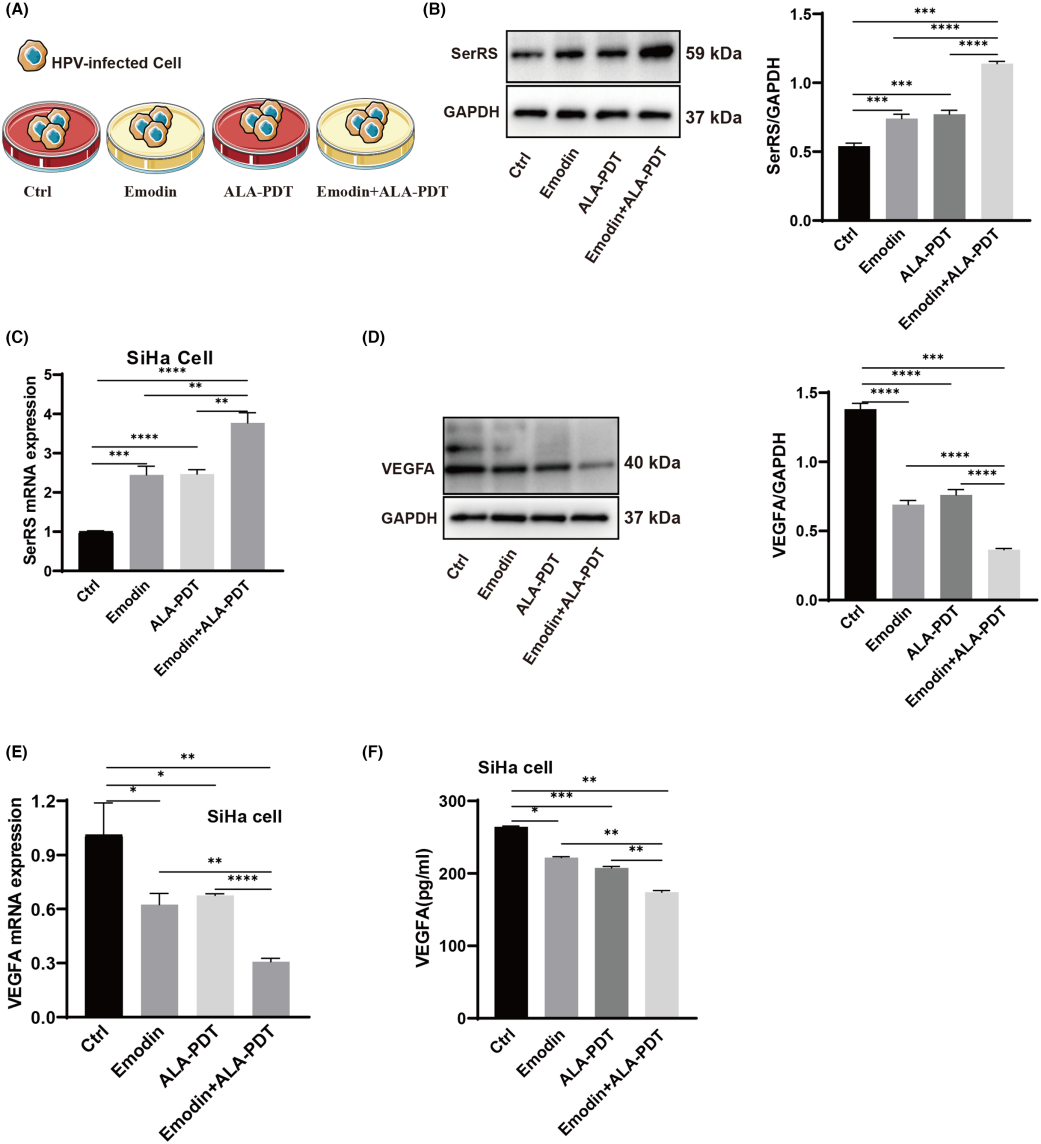
Emodin enhanced the effect of ALA‐PDT through the SerRS‐VEGFA pathway on SiHa cells. (A) Schematic of the experimental design of SiHa cells receiving different treatments. (C, D) Real‐time PCR showed the level of SerRS and VEGFA in SiHa cells which were cultured under different conditions. (B, E) Western blot assay displayed the level of SerRS and VEGFA in SiHa cells which were cultured with emodin and ALA‐PDT or not. (F) ELISA assay showed the secretion of VEGFA in SiHa cells which were divided into different groups. All experimental data were independently repeated for 3 times to obtain the mean ± SD. *p* value, * < 0.05, ** < 0.01, *** < 0.001, **** < 0.0001 by Student's *t* test.

To further explore the molecular mechanism of emodin's enhancement of ALA‐PDT's inhibition of SiHa cell proliferation, we detected expression of SerRS and VEGFA by qPCR and WB. We randomly divided SiHa cells into four groups: medium alone, emodin, ALA‐PDT and emodin combined with ALA‐PDT (Figure 4A). The results showed that emodin combined with ALA‐PDT significantly inhibited VEGFA and promoted SerRS expression compared to ALA‐PDT or emodin monotherapy (Figure 4B–E). ELISA for VEGFA was also performed (Figure 4F). These results indicate that emodin enhanced the efficacy of ALA‐PDT in human papillomavirus‐infected cells through the SerRS‐VEGFA pathway.

### 2.5 Emodin improved the efficiency of ALA‐PDT in vivo by targeting SerRS‐VEGFA pathway

**FIGURE 5:**
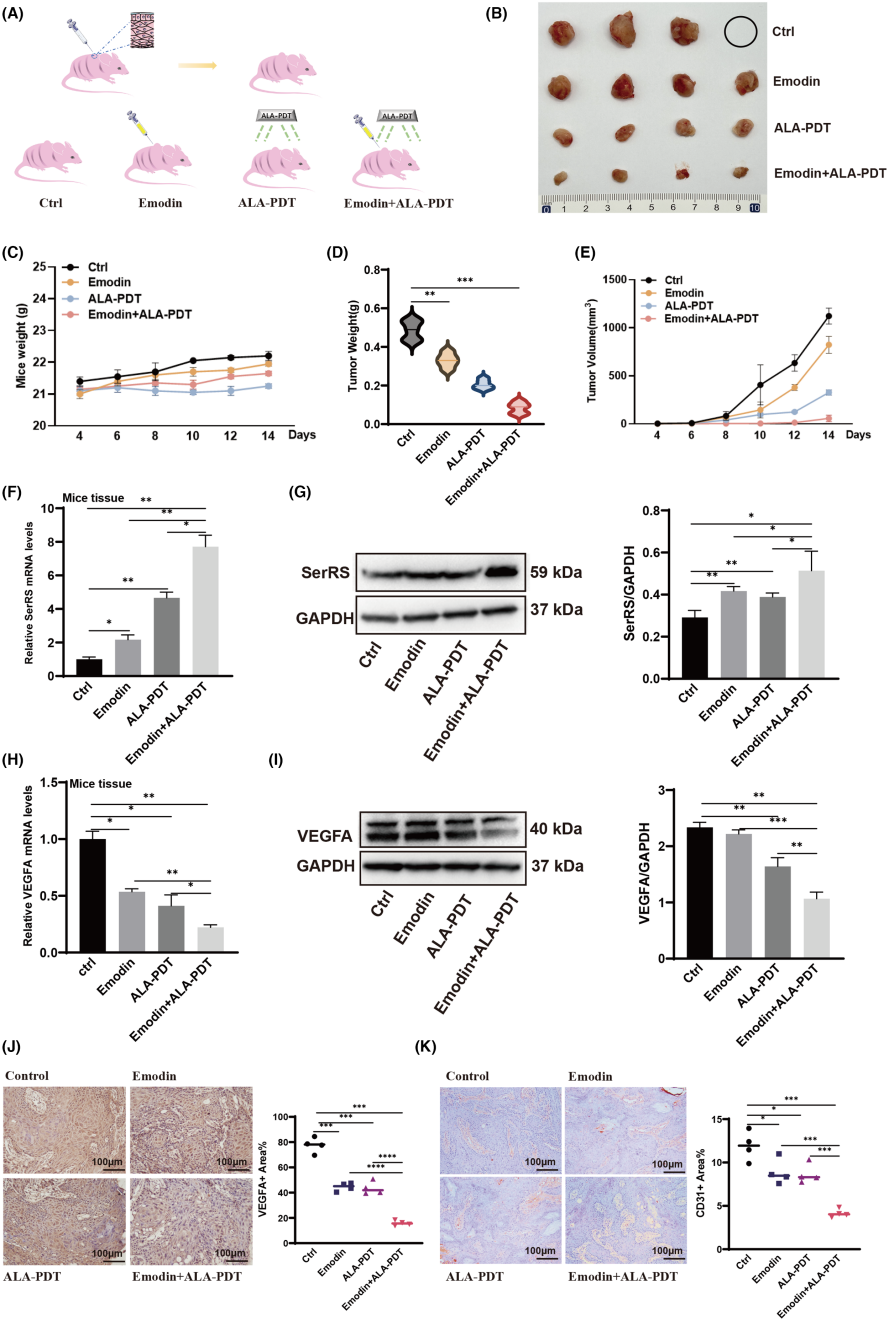
Emodin combined with ALA‐PDT showed a more significant inhibitory effect on proliferation in the xenograft model. (A) Flow diagram of SiHa cells implanted orthotopically into the female BALB/c nude mice and treated intraperitoneally with DMSO, emodin, ALA‐PDT, emodin combined with ALA‐PDT every other day until sacrifice (*n* = 4). (C) The body weights of the mice measured every other day. (B, D, E) Weight and volume of tumours at sacrifice of the mice. (F–I) The expression of SerRS and VEGFA in tumour tissues was analysed by WB and Real‐time PCR. (J, K) The expression of VEGFA and CD31 in tumour tissues was analysed by immunohistochemistry assay (*n* = 4, Student's *t* test). All images were taken at 200× original magnification, Scale bar, 100 μm. All experimental data were independently repeated for 3 times to obtain the mean ± SD. *p* value, * < 0.05, ** < 0.01, *** < 0.001 by Student's *t* test.

To validate emodin's effects in combination with ALA‐PDT on HPV‐related tumours in vivo, we established an animal xenograft model by subcutaneously inoculating SiHa cells into mice and treating them with DMSO, emodin, ALA‐PDT or emodin combined with ALA‐PDT (Figure 5A). Starting from day 4 post‐SiHa cell inoculation, tumour size was measured every other day. The results showed the combination treatment group exhibited more pronounced inhibition of HPV‐infected cell proliferation compared to single‐drug treatment groups (Figure 5B). The body weights of mice across treatment groups did not show significant differences (Figure 5C). On day 14 post‐tumour cell inoculation, mice were euthanized and subcutaneous tumour tissues harvested and weighed. The results demonstrated emodin enhanced ALA‐PDT's inhibitory effect on HPV‐related tumour proliferation. In the combination treatment group, both tumour weight and volume were significantly lower than the control group and single‐drug treatment groups (Figure 5D,E). qPCR and WB were used to assess VEGFA and SerRS expression in tumour tissues across treatment groups. The results confirmed the combination treatment group strongly promoted SerRS and inhibited VEGFA (Figure 5F–I). The results of these animal experiments indicated emodin enhanced ALA‐PDT's effects of promoting SerRS and inhibiting VEGFA in vivo. Immunohistochemistry was used to detect the expression of relevant genes in tumour tissue. The results of VEGFA and CD31 show that emodin significantly increased the inhibitory effect of ALA‐PDT on angiogenesis in vivo (Figure 5J,K).

### 2.6 Emodin improved the efficiency of ALA‐PDT against CA pathological angiogenesis at the tissue level

**FIGURE 6:**
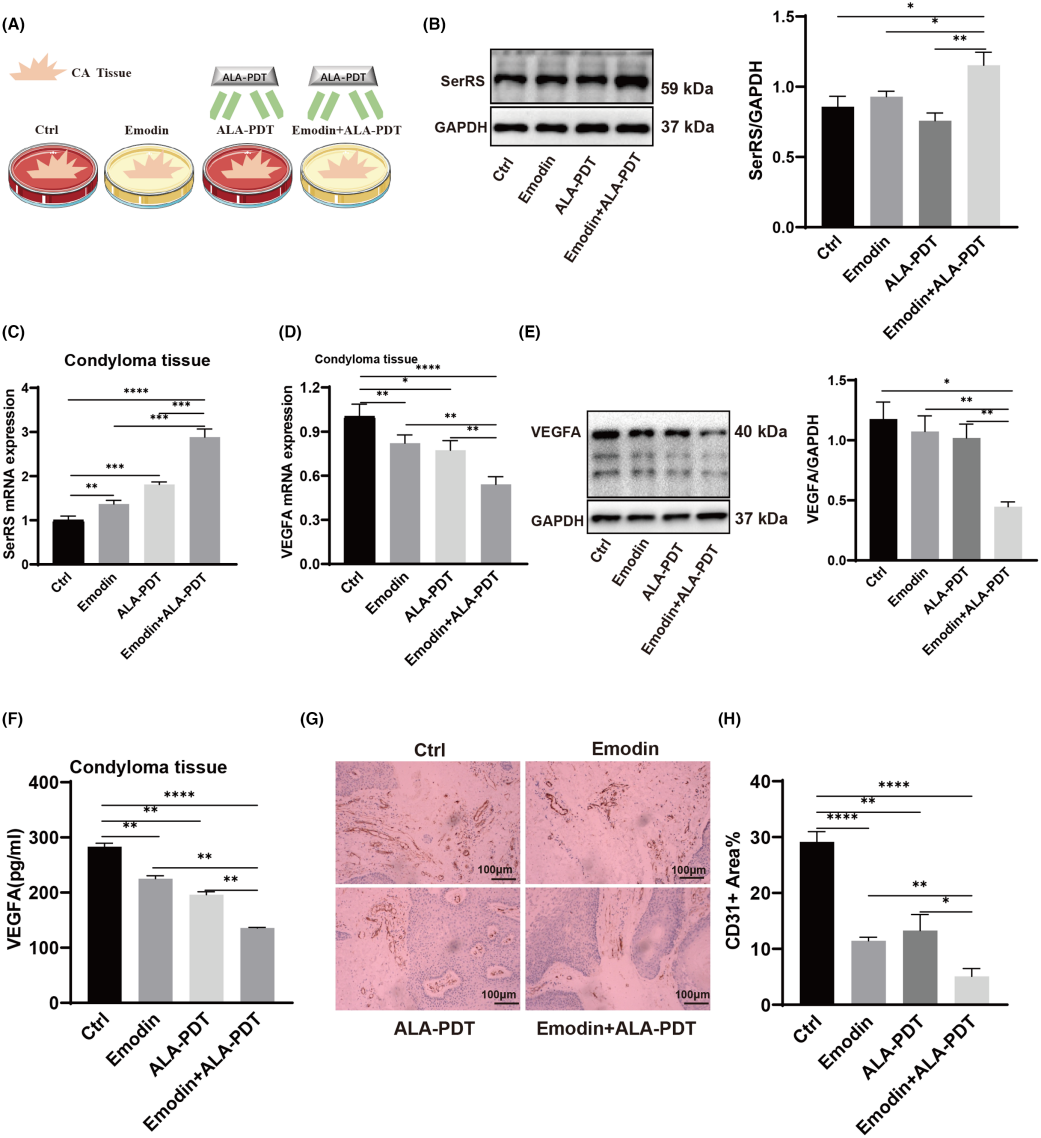
In vivo activity of emodin on condyloma acuminatum (CA) in tissue ex vivo culture model. (A) Diagram of the experimental model of CA tissue ex vivo culture. (C, D) mRNA levels of SerRS and VEGFA in condyloma tissues which were divided equally into four groups as control, emodin, ALA‐PDT and emodin+ALA‐PDT. (B, E) Western blot assay displayed the level of SerRS and VEGFA in condyloma tissues which were divided equally into four groups as control, emodin, ALA‐PDT and emodin+ALA‐PDT. (F) ELISA detection of VEGFA expression in condyloma tissues treated under different conditions. (G, H) Immunohistochemistry assay revealed that the level of CD31 in condyloma tissues which were treated under different conditions. (*n* = 3). All images were taken at 200× original magnification. Scale bar, 100 μm. All experimental data were independently repeated for 3 times to obtain the mean ± SD. *p* value, * < 0.05, ** < 0.01, *** < 0.001, **** < 0.0001 by Student's *t* test.

VEGFA has been reported to promote CA progression. Consistent with our results showing VEGFA upregulation in CA tissue, promoting CA angiogenesis and growth. We hypothesized combined emodin and ALA‐PDT application might greatly inhibit CA progression by targeting the SerRS‐VEGFA pathway suppressing CA angiogenesis. To test this hypothesis, fresh human normal foreskin or CA biopsy samples were utilized for ex vivo cultures examining VEGFA and SerRS expression. VEGFA and SerRS levels were measured by qRT‐PCR, Western blotting and immunohistochemistry in clinical tissues from condyloma acuminatum patients. Tissues were randomly divided into four groups: medium alone, emodin, ALA‐PDT and emodin combined with ALA‐PDT (Figure 6A). Interestingly, expression of VEGFA decreased but SerRS increased in specimens treated with emodin and ALA‐PDT, consistent with SiHa cell experiments. These results indicate emodin improves ALA‐PDT's inhibitory effect on CA tissue through the SerRS‐VEGFA pathway (Figure 6B–E). Additionally, ELISA of VEGFA further confirmed this (Figure 6F). We also used immunohistochemistry to detect changes in CD31 expression levels across treatment groups. Results showed emodin enhanced ALA‐PDT's inhibitory effect on blood vessels in condyloma acuminatum tissue (Figure 6G,H).

## 3 CONCLUSION

Condyloma acuminatum (CA) is a dermatological infectious disease caused by HPV infection, manifesting as proliferative damage to perianal and genital areas. Some scholars indicate CA occurrence and development is closely related to new blood vessel development in focal tissue. VEGFA is a vascular endothelial cell growth factor promoting new blood vessel formation. Clinical studies show VEGFA is significantly overexpressed in CA. We identified an herbal‐derived small molecule emodin with potential to enhance ALA‐PDT therapy for CA by inhibiting VEGFA transcription and angiogenesis in CA through targeting SerRS. Together, our results contribute to a new understanding of CA and provide a promising ALA‐PDT combination therapy inhibiting angiogenesis and CA recurrence by targeting SerRS.

## 4 DISCUSSION

CA is one of the most common sexually transmitted diseases caused by HPV infection.^34^ Not only is the incidence of condyloma acuminatum high, but the recurrence rate after treatment can reach 30%–70%; thus, preventing CA recurrence is more difficult than treating it.^35^ Conventional treatment for CA patients (including freezing, laser, microwave, etc.) mainly focuses on destroying or removing the wart itself, but has difficulty eliminating the virus.^36^ ALA‐PDT is an emerging therapy with high selectivity that has the advantage of effectively eliminating latent and subclinical infection. However, patients undergoing treatment may experience pain, have a long recovery time and cannot avoid recurrence.^37^

The current study found a certain relationship between continuous recurrence of CA and the local focus vascular response with new vessels.^38^ Dermal capillary increase accompanied by lumen dilatation is one of the significant pathological features of CA. Studies have confirmed that the expression level of VEGF in CA skin is significantly higher than that in normal skin.^39^ VEGF plays an important role in promoting dermal angiogenesis in CA. Studies have shown that both mRNA and protein expression of VEGF increase significantly in human foreskin keratinocytes transfected with HPV E6 and E7 oncogenes in vitro.^40^ The HPV E5 oncogene promotes VEGF expression through the epidermal growth factor receptor, MEK/ERK1,2 and PI3K/Akt pathways.^41^ The above studies indicate that VEGF plays a key role in the growth of CA, but the specific mechanism remains to be revealed.

The possible mechanism is that HPV infection induces VEGF production, increasing skin lesion vascular permeability and angiogenesis, resulting in verrucous hyperplastic damage to mucosa and skin. Additionally, VEGF has immunosuppressive properties, and HPV infection can reduce host immunity, particularly cellular immunity. Therefore, VEGF may further aggravate local immunosuppression in skin lesions and promote tissue proliferation.^42^ Consequently, VEGF may be related to CA recurrence to some extent. Further studies showed that the expression of VEGFA in CA tissues was significantly reduced after ALA‐PDT treatment, which may be a possible reason for the inhibition of CA recurrence by ALA‐PDT. However, the specific mechanism of ALA‐PDT inhibiting VEGFA is still unclear.

SerRS is a cytosolic aminoacyl‐tRNA synthetases (aaRS) catalysing serine attachment to tRNA. In particular, SerRS acquired a nuclear localization signal during evolution. Our study showed that SerRS compete with c‐Myc for the promoter region of VEGFA after nuclear translocation, thereby inhibiting VEGFA expression and angiogenesis.^23^ Another study revealed that ATM‐SerRS signalling is a novel mechanism by which UV upregulates VEGFA.^22^ An isoflavone derivative potently inhibits the angiogenesis and the progression of triple‐negative breast cancer by targeting the MTA2/SerRS/VEGFA pathway.^43^ This suggests SerRS may be a new drug target for angiogenesis inhibition. To date, no study has investigated whether SerRS plays a role in the destruction of blood vessels in CA tissues by ALA‐PDT.

In our study, we observed a significant upregulation of VEGFA expression in condyloma acuminata (CA) tissues compared to healthy controls, indicating a potential role of VEGFA in the pathogenesis of CA. Emodin, a natural anthraquinone derivative, demonstrated the ability to inhibit the proliferation of human papillomavirus (HPV)‐infected cells and promote their apoptosis by targeting the SerRS‐VEGFA pathway. This finding suggests that Emodin may disrupt the angiogenic process, which is critical for tumour growth and maintenance. Furthermore, our research revealed that Emodin could enhance the effects of ALA‐PDT by inducing the production of reactive oxygen species (ROS) in SiHa cells, leading to their apoptosis. The synergistic effect of Emodin and ALA‐PDT was then validated at the cellular, murine and human CA tissue levels, highlighting the potential of this combination therapy to improve treatment outcomes for CA.

Another potential cause of CA recurrence is persistent latent HPV infection. Previous research successfully screened emodin, a natural small molecule from traditional chinese medicine, which inhibits VEGFA expression by targeting the transcriptional level.^25^ Additionally, some studies show emodin has broad‐spectrum antiviral activity and inhibits a variety of viruses, but no studies have explored its specific HPV inhibition. This study preliminarily confirmed that emodin enhances ALA‐PDT's inhibition of CA proliferation by promoting SerRS expression and inhibiting angiogenesis. In follow‐up, we will focus on whether emodin has targeted HPV inhibition. To provide a theoretical basis for enhancing ALA‐PDT therapeutic effects and preventing CA recurrence.

This study first demonstrated in HPV‐infected cells and nude mice bearing SiHa cells that specifically promoting SerRS expression can inhibit angiogenesis and even cell proliferation via transcriptional inhibition of VEGFA. Therefore, SerRS may be a new target for CA therapy. In addition, lacking a standard animal model of CA, this project used ex vivo culture of CA tissues. Previous studies confirmed CA tissues of patients can be cultured ex vivo for 72 h or longer,^44^ better simulating clinical CA patients receiving ALA‐PDT treatment. However, this experimental model omits the systemic changes and material exchange that would normally occur. Therefore, future studies should endeavour to construct superior disease models, for more accurately exploring ALA‐PDT's basic principles in CA treatment.

This study demonstrated for the first time that the small molecule monomer emodin can not only specifically promote SerRS expression and inhibit VEGFA but may also have antiviral effects. In clinical practice, emodin combined with ALA‐PDT can be used to treat CA more effectively. However, to date, data on the bioavailability, pharmacokinetics and metabolism of emodin are limited. Whether emodin affects the metabolism of other pharmacological agents needs to be evaluated before it can be safely used in combination with other medications. Therefore, more work is needed to improve the bioavailability of emodin, extend the in vivo action time of emodin and give emodin targeting ability to achieve enhanced efficacy while reducing toxic side effects.

The novelty of our study lies in the discovery that the combination of Emodin and ALA‐PDT can suppress angiogenesis in CA by upregulating the expression of SerRS, thereby inhibiting VEGFA expression. This dual‐targeting approach not only enhances the therapeutic efficacy of ALA‐PDT but also provides a new perspective on the management of CA by targeting both the viral infection and the angiogenic process.

## 5 MATERIALS AND METHODS

### 5.1 Patient specimens

The study involved collecting five specimens from patients with condyloma acuminatum and five normal preputial tissue specimens from January to March 2023 at Nanfang Hospital of Southern Medical University in Guangzhou, China. All human specimen studies were approved by Nanfang Hospital, adhering to ethical guidelines.

### 5.2 Chemicals

Emodin, an important rhubarb monomer and active ingredient, was obtained from Dalian Meilun Biotechnology Co. Ltd. (MCE, HY‐14393) and dissolved in dimethyl sulfoxide. ALA was acquired from Fudan Zhangjiang Biopharmaceutical Co. Ltd., Shanghai, China, and dissolved with PBS (phosphate buffer saline). All chemicals were analytical grade.

### 5.3 Cell cultures

SiHa cells were obtained from the Clinical Experimental Center of Nanfang Hospital, Southern Medical University in Guangzhou, China. Prior to use, these cells were screened to ensure freedom from mycoplasma infection. The cell culture medium consisted of DMEM (Dulbecco's modified Eagle medium) supplemented with 10% fetal bovine serum, 100 U/mL penicillin and 100 mg/mL streptomycin. The cells were cultured under optimal conditions in a humidified incubator maintained at 37°C with a 5% CO_2_ atmosphere.

### 5.4 Cell viability assays

SiHa cells were seeded in 96‐well plates at 6 × 10^3^ cells per well and allowed to adhere for 24 h. Cells were then treated with various concentrations of emodin‐containing medium and incubated at 37°C for 24, 48 or 72 h in the dark. CCK‐8 reagent (10 μL/well) was added to the culture medium, and the plates were incubated at 37°C for 3 h. Optical density (OD) at 450 nm was measured using a microplate reader. Relative cell viability (%) was calculated by dividing the OD of the experimental wells by the OD of the control wells, subtracting the blank OD and multiplying by 100.

### 5.5 In‐vitro photodynamic therapy

SiHa cells were incubated with different concentration of 5‐aminolevulinic acid (0.25, 0.5, 1.0 and 2.0 mmol/L) for 12, 18, 24, 30, 36 and 42 h. The fluorescence intensity of PpIX was measured by the BMG multifunctional enzyme marker. Thereafter, based on changes in PpIX fluorescence intensity in the SiHa cells after different time periods, the optimal ALA concentration and incubation time range was identified. Furthermore, the SiHa cells were pre‐treated with ALA at 0.25 mmol/L for 24 h. Then we treated the cells with PDT (635 nm wavelength red light, illumination distance 10 cm, power 60 mW/cm^2^) at energies of 0, 0.6, 1.2, 2.4, 4.8 and 6.0 J/cm^2^, and cell viability was measured using the CCK‐8 assay.

### 5.6 Flow cytometry analysis

For apoptosis analysis, SiHa cells were detected using the Annexin V‐APC/PI apoptosis kit (MULTI SCIENCES). Briefly, SiHa cells were subjected to PDT treatment and then incubated in complete DMEM containing emodin after being cultured with ALA for 24 h. After 24 h, the cells were rinsed with PBS and suspended in binding buffer before being subjected to staining with annexin V‐APC and PI in the absence of light for a duration of 5 min. The flow cytometer from BD Biosciences was utilized to determine the percentage of cells positive for annexin V.

To detect intracellular reactive oxygen species (ROS), SiHa cells were plated in 6‐well plates and adhered for 24 h. They were then subjected to different treatments. The cells were probed with serum‐free medium containing diluted 2′,7′‐dichlorofluorescein (DCFH‐DA) for 20 min in a humidified incubator maintained at 37°C with a 5% CO_2_ atmosphere in the dark, with mixing every 3–5 min. The cells were rinsed with serum‐free medium and trypsinized for analysis. The flow cytometer with an excitation wavelength of 488 nm and an emission wavelength of 525 nm was employed to measure the percentage of cells positive for ROS.

### 5.7 Western blot analysis

Cells and tissues were lysed using RIPA lysis buffer containing protease inhibitor, phosphatase inhibitor cocktail, per manufacturer's specifications. Protein concentration was determined by BCA protein assay kit (Beyotime, BL521A, EH). Equivalent total protein amounts were separated by 8%–10% SDS‐PAGE, then transferred onto polyvinylidene difluoride (PVDF) membranes. Following overnight incubation with primary antibodies at 4°C, secondary antibodies were applied. Primary antibodies used were rabbit anti‐human VEGFA (ab214424, abcam, American), SerRS (PAB34791‐50, Bioswamp, China), Bcl2 (68103‐1‐Ig, Proteintech, China), Bax (60267‐1‐Ig, Proteintech, China) and GAPDH (#5174S, Cell Signalling Technology, America). Secondary antibody was HRP‐conjugated goat anti‐rabbit (SA00001‐2, proteintech, China). Protein bands were analysed using Image Lab analysis software from Bio‐Rad, CA, USA.

### 5.8 Quantitative PCR assay

Total RNAs of SiHa cells and condyloma acuminata tissues were extracted using TRIzol (Invitrogen) and were reversely transcribed into first‐strand cDNA using All‐in‐one First Strand cDNA Synthesis Kit II for qPCR (with dsDNase) (SEVEN, Beijing, China). We utilized the 2x SYBR Green qPCR Master Mix (SEVEN, Beijing, China) for conducting quantitative RT‐PCR on Light Cycler 96 (Roche). The obtained results were normalized with respect to ACTB and expressed as the fold change of individual mRNA transcripts. The expression level of mRNAs was determined using 2^−△△Ct^. Here are the primer sequences employed for quantitative real‐time PCR:

ACTB‐h‐F:5′‐AGCGAGCATCCCCCAAAGTT‐3′;

ACTB‐h‐R:5′‐GGGCACGAAGGCTCATCATT‐3′;

VEGFA‐h‐F:5′‐GAGGGCAGAATCATCACGAAG‐3′;

VEGFA‐h‐R:5′‐TGTGCTGTAGGAAGCTCATCTCTC‐3′;

SerRS‐h‐F:5′‐AAGAAAGCAGCAGCAAGAGACG‐3′;

SerRS‐h‐R:5′‐CATGCGAGGAGACAGGAACATC‐3′.

### 5.9 Plasmid construction and establishment of stable cell lines

DNA oligonucleotide encoding shRNA against human SerRS (5′‐GGCATAGGGACCCATCATTGA‐3′ in 3′UTR) was inserted into the pLentiLox‐hH1 plasmid, modified from the pLentiLox 3.7 plasmid to contain an H1 promoter (between Xba I and Xho I sites) to drive the shRNA expression. For nontargeting control shRNA, we used the sequence 5′‐TAAGGCTATGAAGAGATAC‐3′. The lentiviruses were produced in 293T cells according to the manufacturer's instructions. Then, the cells were infected with lentivirus for 24 h and cultured for 48 h, followed by selection using 2.5 μg/mL puromycin. The cells infected with shRNA lentiviruses were subjected to analysis such as real‐time quantitative reverse transcription PCR (qRT‐PCR) and Western blot.

### 5.10 Enzyme‐linked immunosorbent assay (ELISA)

Supernatants from SiHa cells incubated under different conditions were collected. ELISA was used for the detection and quantification of secreted VEGFA following the manufacturer's specifications. The signal was identified at a wavelength of 450 nm (DLDEVELOP, Wuxi, China).

### 5.11 Tissue ex vivo culture

Clinical specimens of excised residual tissue from patients with condyloma acuminatum were collected for pathological examination. The wart tissues were rinsed three times with pre‐chilled PBS containing 3% gentamicin/penicillin for 5 min each time to remove blood contamination and clear subcutaneous tissue. The cleaned wart tissues were then longitudinally incised from the epidermis to the dermis and divided into four (or more) equal parts. These parts were grouped according to different treatment methods, such as the DMSO group, the Emodin group, the ALA‐PDT group (0.25 mmol/L ALA for 24 h followed by 1.2 J/cm^2^ PDT, 635 nm wavelength red light, illumination distance 10 cm, power 60 mW/cm^2^) and the Emodin+ALA‐PDT (20 μmol/L Emodin for 24 h followed by ALA‐PDT) group, and placed in culture dishes containing culture medium. The dermal side was submerged in the culture medium, while the epidermal side was exposed to air. The samples were cultured in a constant temperature incubator at 37°C with 5% CO_2_. Previous studies have demonstrated that fresh tissues maintained their viability in vitro for up to 72 h. In this study, total RNA or protein was extracted from the experimental groups after 12 h of treatment, or they were fixed with 10% formalin for an immunohistochemical (IHC) experiment.

### 5.12 Animal studies

This study was approved by the Animal Research Committee of Southern Medical University. Our BALB/c nude mice (6–8 weeks old) were obtained from Guangdong Experimental Animal Center (Guangzhou, China) and were housed in pathogen‐free animal facilities with 12‐h light/dark cycles. For xenograft mouse models, SiHa cells (1 × 10^7^) were injected subcutaneously into the right buttock of the mice. After tumours became palpable, the mice were randomly divided into four groups and received an intraperitoneal injection of DMSO, emodin, ALA‐PDT or emodin combined with ALA‐PDT every other day. Tumour volume (V) was measured every other day using callipers and calculated using the standard formula: V = length × width^2/2.

### 5.13 Immunohistochemical analysis

Immunohistochemical analysis of VEGFA (1:50; abcam) and CD31 (1:100; abcam) primary antibodies in CA tissues, normal preputial tissues and CA tissues subjected to ex vivo culture was conducted. Briefly, tissue specimens were paraffin‐embedded and fixed in 10% formalin. Following deparaffinization and rehydration of 5 μm tissue sections, antigen retrieval was performed by boiling in citrate buffer solution prior to immunostaining. To block non‐specific binding, 10% goat serum in PBS was used, followed by incubation with a rabbit anti‐human VEGFA and CD31 antibody at 4°C overnight. The nuclei were labelled with DAPI. A Nikon Eclipse Ti microscope was used to view the slides, and areas of immune positive cells were quantified using Image J software version 1.50 from National Institutes of Health, Bethesda, MD, USA. The same exposure conditions were used for all images. The positive signal is brownish yellow or brownish brown. Area% represents the positive signal area as a percentage of the total area.

### 5.14 Statistical analysis

Means ± standard deviations (depicted by error bars) were utilized to represent experimental data, and statistical analysis was executed by implementing Student's *t*‐test or analysis of variance. The software utilized for all statistical analyses was GraphPad Prism 8. For statistical significance, *p* < 0.05 was taken into consideration.

## AUTHOR CONTRIBUTIONS

**Hongyan Lu:** Data curation (equal); writing – original draft (equal). **Zhangsong Peng:** Conceptualization (equal); formal analysis (equal). **Yingrui Luo:** Data curation (equal); methodology (equal). **Zhaohui Zheng:** Investigation (equal); software (equal). **Changxing Li:** Project administration (equal); resources (equal). **Qi Wang:** Formal analysis (equal); investigation (equal). **Chao Han:** Software (equal); visualization (equal). **Youyi Wang:** Methodology (equal); resources (equal). **Liuping Liang:** Supervision (equal); validation (equal). **Kang Zeng:** Funding acquisition (equal); project administration (equal); writing – review and editing (equal). **Yuxiang Chen:** Supervision (equal); writing – review and editing (equal).

## FUNDING INFORMATION

The National Natural Science Foundation of China (NO.82073464), Guangzhou Science and technology planning project (NO.202201011452), The 73rd batch of General projects of China Postdoctoral Science Foundation (R11001010), GuangDong Basic and Applied Basic Research Foundation (2023A1515110487; 2023A1515110981) and the President Foundation of Nanfang Hospital, Southern Medical University (2021B004; 2021 L002) all supported this study.

## CONFLICT OF INTEREST STATEMENT

No conflict of interest is disclosed by the authors.

## Data Availability

The published article included all data generated or analysed during this study.
